# Prevalence of Associated Extraoral Symptoms and Comorbidities in Burning Mouth Syndrome Patients: A Systematic Review

**DOI:** 10.1111/odi.70144

**Published:** 2025-11-15

**Authors:** João Paulo Gonçalves de Paiva, Caique Mariano Pedroso, Rosa María López‐Pintor Muñoz, Milda Chmieliauskaite, Alessandro Villa, Jacks Jorge, Alan Roger Santos‐Silva

**Affiliations:** ^1^ Departamento de Diagnóstico Oral, Faculdade de Odontologia de Piracicaba Universidade Estadual de Campinas São Paulo Brazil; ^2^ Department of Dental Clinical Specialties, ORALMED Research Group Complutense University of Madrid Madrid Spain; ^3^ Departament of Oral Medicine, School of Dentistry University of Washington Seattle Washington USA; ^4^ Oral Medicine, Oral Oncology and Dentistry, Miami Cancer Institute, Baptist Health South Florida Miami Florida USA

**Keywords:** burning mouth syndrome, medically unexplained symptoms, prevalence

## Abstract

**Objective:**

Patients with burning mouth syndrome (BMS) suffer from oral pain in the absence of oral lesions. Although less frequently, they may also experience extraoral symptoms that are not readily apparent to clinicians. This study aimed to systematically assess the prevalence of associated extraoral symptoms and comorbid disorders in BMS patients.

**Methods:**

Comprehensive searches were performed in five electronic databases (PubMed, Web of Science, Scopus, Embase, and LILACS). Observational studies that reported extraoral symptoms in primary BMS patients were included. The collected data were analyzed descriptively. The quality assessment of the included studies was conducted using the Joanna Briggs Institute tools.

**Results:**

We included 22 studies encompassing 2786 BMS patients. Most patients were female (*n* = 2138; 76.7%). BMS‐associated extraoral symptoms and comorbidities disorders included sleep disturbances (71.8%), anxiety (65.7%), depression (60.6%), alexithymia (6.1%), tinnitus (5.9%), low back pain (45.3%), and headache (4.4%). Fifteen studies were classified as low risk, six as moderate risk, and one as high risk of bias.

**Conclusions:**

BMS patients experience associated extraoral symptoms and comorbid disorders in addition to intraoral pain. These symptoms must be taken into account by clinicians and receive appropriate clinical treatment, as they can significantly affect patients’ quality of life.

## 1 Introduction

Highly prevalent diseases present signs and symptoms that are readily identified, interpreted, and contextualized by healthcare professionals. Rare diseases, conversely, often present poorly defined clinical features, leading to diagnostic delays and suboptimal management (Joachim and Acorn 2003). This frequently results in patients feeling their complaints are neglected, perceiving their symptoms as “invisible” (Brennan and Creaven 2016; Ciribassi and Patil 2016).

The term “invisible symptoms” refers to symptoms that are often overlooked by clinicians yet significantly impair patients’ quality of life. This phenomenon has been well documented in chronic conditions such as systemic lupus erythematosus, multiple sclerosis, sickle cell anemia, and scleroderma (Brennan and Creaven 2016; Ciribassi and Patil 2016; Joachim and Acorn 2003; Parker et al. 2021).

Burning mouth syndrome (BMS) is a chronic pain disorder affecting 1.73% of the general population (de Lima‐Souza et al. 2024; Wu et al. 2022). It is defined by the International Classification of Orofacial Pain (ICOP) as a persistent burning or dysesthetic sensation, lasting at least 2 h per day and recurring for a minimum of 3 months, often accompanied by somatosensory alterations. This occurs in the absence of any clinically identifiable oral lesions or systemic conditions (International Classification of Orofacial Pain, 1st edition (ICOP)’ 2020). The International Headache Society (IHS, ICDH‐3) criteria differentiate primary BMS, which occurs in the absence of local or systemic factors, from secondary BMS, which is linked to identifiable local causes or systemic disorders (‘Headache Classification Committee of the International Headache Society (IHS) The International Classification of Headache Disorders, 3rd edition’ 2018; International Classification of Orofacial Pain, 1st edition (ICOP)’ 2020).

In BMS, extraoral symptoms are frequently perceived as “invisible” by patients because they are often overlooked by clinicians at all stages of care, from prediagnostic evaluation through long‐term management. This neglect includes both initially missed symptoms and ongoing comorbid manifestations that receive little attention in treatment plans, contributing to psychological distress and reducing patients' quality of life (de Lima‐Souza et al. 2024; Freilich et al. 2020; Mignogna et al. 2011; Santos‐Silva and Villa 2024).

This perception of invisibility arises when some patient complaints are categorized as medically unexplained symptoms, defined as persistent physical or psychological complaints without a definitive diagnosis despite comprehensive assessment, or when they are not recognized as legitimate manifestations of BMS. Although no organic cause can be identified, these symptoms represent genuine experiences that profoundly impair daily functioning and quality of life (Husain and Chalder 2021).

Therefore, comprehensive BMS patient management requires a more holistic approach that includes the identification and treatment of all associated and underrecognized complaints (Santos‐Silva and Villa 2024). Given the limited understanding of these associated extraoral symptoms and comorbid disorders in BMS, this study aimed to investigate their prevalence and clinical relevance in this patient population.

## 2 Methods

### 2.1 Eligibility Criteria

This systematic review was structured according to the PECOS framework (Schardt et al. 2007). The population (P) included adult patients (≥ 18 years). The exposure (E) consisted of BMS patients diagnosed with primary BMS according to the ICOP and/or IHS (ICDH‐3) criteria. A comparator (C) was not applicable because we only wanted to analyze these alterations in patients with BMS. The outcomes (O) of interest were associated with extraoral symptoms and comorbid disorders described in BMS patients. Study design (S) included case–control, cross‐sectional, cohort, clinical trials, and case series studies.

We included studies published in English or Spanish that enrolled at least 10 patients and reported extraoral symptoms in BMS patients. We selected studies adhering to the International Classification of Headache Disorders (ICHD‐3) (‘Headache Classification Committee of the International Headache Society (IHS) The International Classification of Headache Disorders, 3rd edition’, 2018) or International Classification of Orofacial Pain (ICOP) (‘International Classification of Orofacial Pain, 1st edition (ICOP)’, 2020) classification criteria. We selected studies reporting at least one associated extraoral symptom and comorbid disorder expressed as absolute or relative frequencies.

We excluded studies that did not use IHS or ICOP 2020 criteria, studies reporting associated extraoral symptoms and comorbidities only as means and standard deviations, review articles, conference abstracts, letters to the editor, and case reports.

### 2.2 Information Sources and Search Strategy

A comprehensive search was conducted in February 2025 and subsequently updated in August 2025 across five electronic databases: PubMed, Scopus, Web of Science, Embase, and LILACS, with no restrictions on publication dates. Additional searches were conducted in the gray literature sources, including ProQuest and Google Scholar. Manual searches of the reference lists from included articles were also performed to identify publications that were eventually missed by electronic searches. The complete search strategy for all databases is provided in Table S1.

### 2.3 Selection Process

Two independent reviewers (J.P.G.P. and C.M.P.) screened all titles and abstracts retrieved through the initial search. Studies meeting the predefined eligibility criteria at the title/abstract stage underwent full‐text evaluation. The same reviewers then assessed these selected studies in full‐text format, including those identified through manual reference searches. Disagreements between reviewers were resolved through consensus‐based discussion, with unresolved cases arbitrated by a third author (A.R.S.S.). References and duplicates were managed using the Rayyan platform (https://www.rayyan.ai).

### 2.4 Data Collection Process and Data Items

Two authors (J.P.G.P. and C.M.P.) independently extracted data using Microsoft Excel (version 2203). The following variables were extracted: study author, publication year, country, study design, diagnostic criteria for BMS, total sample size, BMS sample gender, patients’ age (minimum, maximum, mean, and standard deviation [SD]), habits, number of patients with depression, anxiety, and sleep disturbances, and number and type of other BMS‐associated extraoral symptoms and comorbid disorders. Discrepancies between reviewers were resolved through initial discussion, with unresolved cases referred to a third author (A.R.S.S.) for arbitration.

### 2.5 Risk of Bias Assessment

The methodological quality and risk of bias of included studies were evaluated using the Joanna Briggs Institute (JBI) critical appraisal tools for observational studies (Moola et al. 2020). Each appraisal item was independently assessed by two authors, as recommended, and classified as “yes,” “no,” “unclear,” or “not applicable”. Studies were categorized by overall risk of bias based on the percentage of “yes” responses: studies scoring > 70% were classified as low risk, those scoring 50%–70% as moderate risk, and studies scoring < 50% as high risk of bias.

### 2.6 Effect Measures

The primary outcome of this systematic review was to assess the prevalence of associated extraoral symptoms and comorbid disorders in BMS patients. Data were presented as both absolute and relative frequencies (percentages).

### 2.7 Synthesis Methods

Descriptive statistics were conducted to summarize and interpret the findings of the included studies. The prevalence of extraoral symptoms/comorbidities was calculated based on the number of patients reporting each symptom and the total number of BMS patients assessed for that symptom. A meta‐analysis was not feasible to conduct due to the limited number (*n* = 3) and heterogeneity of cross‐sectional studies included in this systematic review. Specifically, one study did not report quantitative data on extraoral symptoms such as depression, anxiety, and sleep disorders. Another failed to report other extraoral symptoms, and the last had a small sample size, which contributed to high heterogeneity in the results.

## 3 Results

### 3.1 Study Selection

**FIGURE 1:**
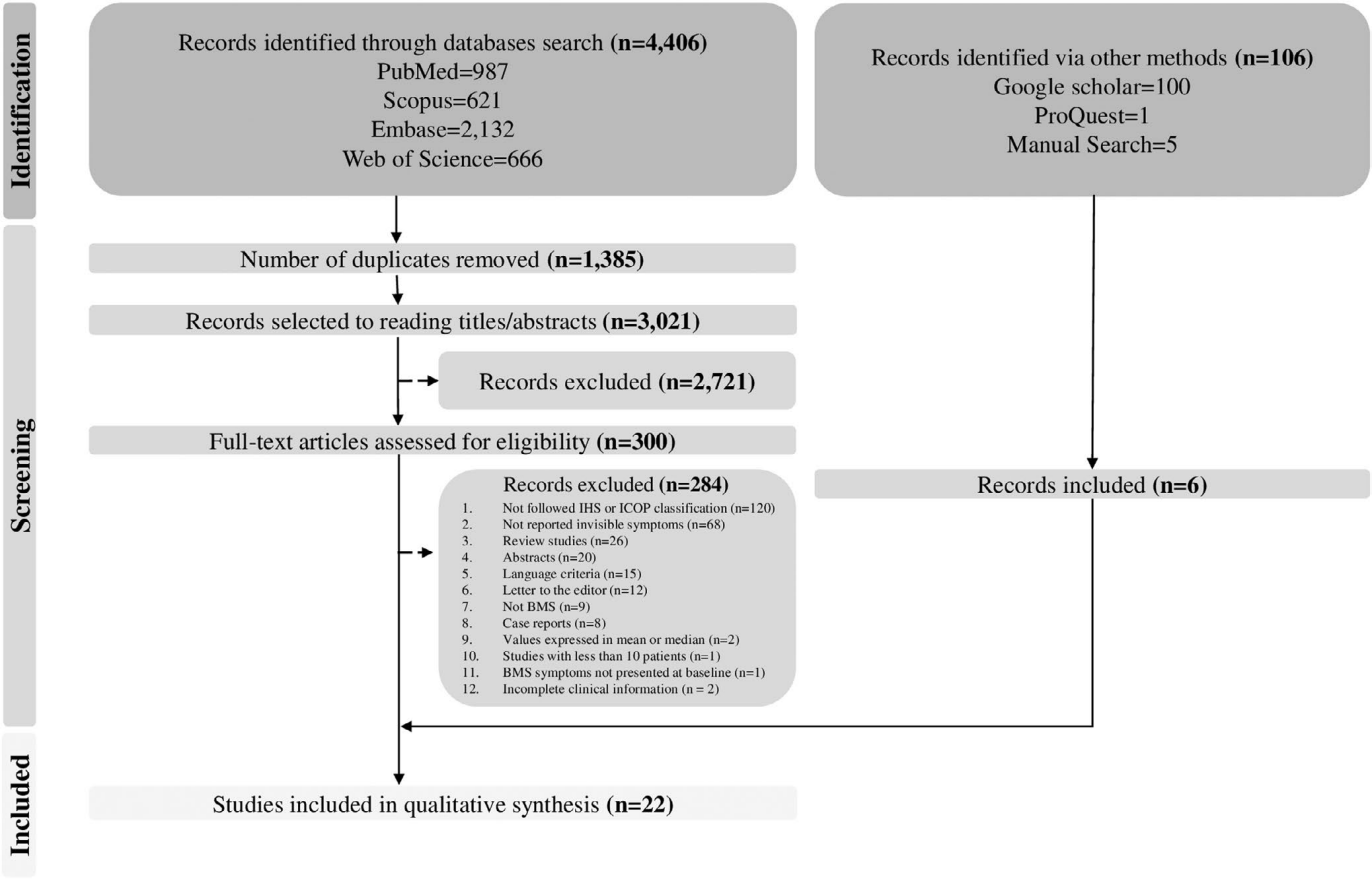
Flowchart describing the selection process of included studies in this systematic review.

Figure 1 illustrates the PRISMA flowchart detailing the study selection process. Electronic searches across scientific databases retrieved 4406 studies. After removing 1385 duplicates, 3021 studies underwent title and abstract screening. This initial screening excluded 2721 studies for not meeting the eligibility criteria, leaving 300 studies for full‐text review. Full‐text assessment excluded 284 studies for not meeting the eligibility criteria, resulting in 16 studies. An additional six articles retrieved from the gray literature and manual search were included, yielding a total of 22 studies for qualitative synthesis.

### 3.2 Study Characteristics

Among 22 articles, 13 were case–control studies, followed by eight cross‐sectional studies, and one cohort study encompassing a total of 2786 BMS patients. The studies were published between 2012 and 2025. Studies were predominantly conducted in Italy (*n* = 10; 45.4%), the United States of America (*n* = 3; 13.6%), and South Korea (*n* = 2; 10%). Brazil, China, England, Iran, Japan, Romania, and Spain accounted for one study each (5%) (Tables S1 and S2).

Among the total sample of 2786 BMS patients, diagnostic criteria included the ICOP criteria (*n* = 1852; 66.5%) or the IHS criteria (*n* = 934; 33.5%) (Table 1). Female patients were more frequent (*n* = 2138; 76.7%) than males (*n* = 648; 23.3%). Patient ages ranged widely from 21 to 85 years (mean age: 63.15 ± 8.48 years). Habits were reported for 1922 patients, of which 335 (17.4%) were smokers and 356 (18.5%) were alcohol users (Table 1).

**TABLE 1:** Clinical and demographic features, depression, anxiety, and sleep disturbances assessment in BMS patients.

Variable	*n* (%)
BMS patients	2786
Female	2138 (76.7)
Male	648 (23.3)
ICOP	1852 (66.5)
IHS (ICDH‐3)	934 (33.5)
Age range	21–85 years
Mean age (SD)	63.15 ± 8.48
Habits	1922 (100)
Smokers	335 (17.43)
Alcohol users	356 (18.52)
Sample/prevalence	***n* (%)**
Depression (*n* = 20)	
Questionnaire/scale	
Hamilton Rating Scale for Depression (HAM‐D)	9 (45)
Not specified	4 (20)
Beck Depression Inventory (BDI)	1 (5)
Depression Anxiety Stress Scales‐21 (DASS‐21)	1 (5)
Diagnostic and Statistical Manual of Mental Disorders (DSM‐IV‐TR)	1 (5)
Hospital Anxiety and Depression Scale (HADS)	1 (5)
International Classification of Diseases (ICD‐11)	1 (5)
MINI‐PLUS	1 (5)
Montgomery and Asberg Depression Rating Scale (MADRS)	1 (5)
Total sample of depression‐assessed BMS patients	2057 (100)
Depression prevalence among BMS patients	1246 (60.6)
Anxiety (*n* = 19)	
Questionnaire/scale	
Hamilton Rating Scale for Anxiety (HAM‐A)	8 (41.11)
Not specified	4 (21.05)
Hospital Anxiety and Depression Scale (HADS)	3 (15.79)
Depression Anxiety Stress Scales‐21 (DASS‐21)	1 (5.26)
International Classification of Diseases (ICD‐11)	1 (5.26)
MINI‐PLUS	1 (5.26)
State–Trait Anxiety Inventory (STAI)	1 (5.26)
Total sample of anxiety‐assessed BMS patients	1989 (100)
Anxiety prevalence among BMS patients	1307 (65.7)
Sleep disturbances (*n* = 13)	
Questionnaire/scale	
Pittsburgh Sleep Quality Index (PSQI)	9 (69.23)
Not specified	2 (15.38)
Insomnia Severity Index (ISI)	1 (7.69)
Symptom Checklist‐90‐Revised (SCL‐90R)	1 (7.69)
Total sample of sleep‐quality assessed BMS patients	2353 (100)
Sleep disturbances prevalence among BMS patients	1690 (71.8)

Abbreviations: BMS, burning mouth syndrome; SD, standard deviation.

Table 1 provides data on the assessment of depression, anxiety, and sleep disturbances in BMS patients included in this study. Depression was assessed in 20 studies (2057 patients) using eight different questionnaires. Most studies employed the Hamilton Rating Scale for Depression (HAM‐D) (*n* = 9; 45%). One study each (5%) used the following: Beck Depression Inventory (BDI), Depression Anxiety Stress Scales‐21 (DASS‐21), Diagnostic and Statistical Manual of Mental Disorders (DSM‐IV‐TR), Hospital Anxiety and Depression Scale (HADS), International Classification of Diseases (ICD‐11), Mini‐International Neuropsychiatric Interview with broader diagnostic coverage (MINI‐PLUS), or Montgomery and Asberg Depression Rating Scale (MADRS). Four studies (20%) reported prevalence rates without specifying diagnostic criteria. Among all BMS patients assessed for depression, the prevalence was 60.6% (*n* = 1246).

Anxiety was assessed in 19 studies (1989 patients) using six different questionnaires. Most studies used the Hamilton Rating Scale for Anxiety (HAM‐A) (*n* = 8; 41.1%), or the Hospital Anxiety and Depression Scale (HADS) (*n* = 3; 15.8%). One study each (5.3%) used the Depression Anxiety Stress Scales‐21 (DASS‐21), the International Classification of Diseases (ICD‐11), MINI‐PLUS, or the State–Trait Anxiety Inventory (STAI). Four studies (21%) did not specify the tool used. Prevalence of anxiety among assessed patients was 65.7% (*n* = 1307).

Sleep quality was investigated in 13 studies (2353 patients). Questionnaires for sleep disturbances assessment included the Pittsburgh Sleep Quality Index (PSQI) (*n* = 9; 69.2%), Insomnia Severity Index (ISI) (*n* = 1; 7.7%), and Symptom Checklist‐90‐Revised (SCL‐90R) (*n* = 1; 7.7%). Two studies (15.4%) did not specify the tool used. Among the assessed patients, the prevalence of sleep disturbances was 71.8% (1690 patients).

### 3.3 Results of Individual Studies

The complete dataset is provided in Table S2. The sample size of patients included in the selected studies ranged from 25 to 583. Depression status was assessed in 20 studies, anxiety in 19 studies, and sleep disturbances in 13 studies, using different tools. Among studies examining depression and anxiety, two failed to report the prevalence of these conditions in absolute or relative values, making it impossible to include them in our analysis. Other BMS‐associated extraoral symptoms and comorbid disorders were assessed in seven studies and comprised both psychological/somatic and physical symptoms.

### 3.4 Risk of Bias

A critical appraisal of the 13 case–control studies included in this systematic review (Table S3) revealed that 10 studies (76.9%) were classified as having a low risk of bias, while three (23.1%) demonstrated a moderate risk. The most frequent methodological limitations in these studies involved inadequate matching approaches and insufficient strategies to address confounding factors. Among the eight cross‐sectional studies (Table S3), five studies (62.5%) were categorized as low risk, and three (37.5%) achieved moderate risk classification. The most frequently observed limitations were related to inadequate strategies for addressing confounding factors and insufficient statistical analysis. The single cohort study included in this systematic review was classified as high risk of bias (Table S3). Specifically, the study lacked a defined comparison group and strategies to address confounding factors. The follow‐up time was unreported, the proportion of patients lost to follow‐up was not described, strategies to address incomplete follow‐up were not utilized, and the statistical analysis employed was inadequate.

### 3.5 Synthesis of Results

**FIGURE 2:**
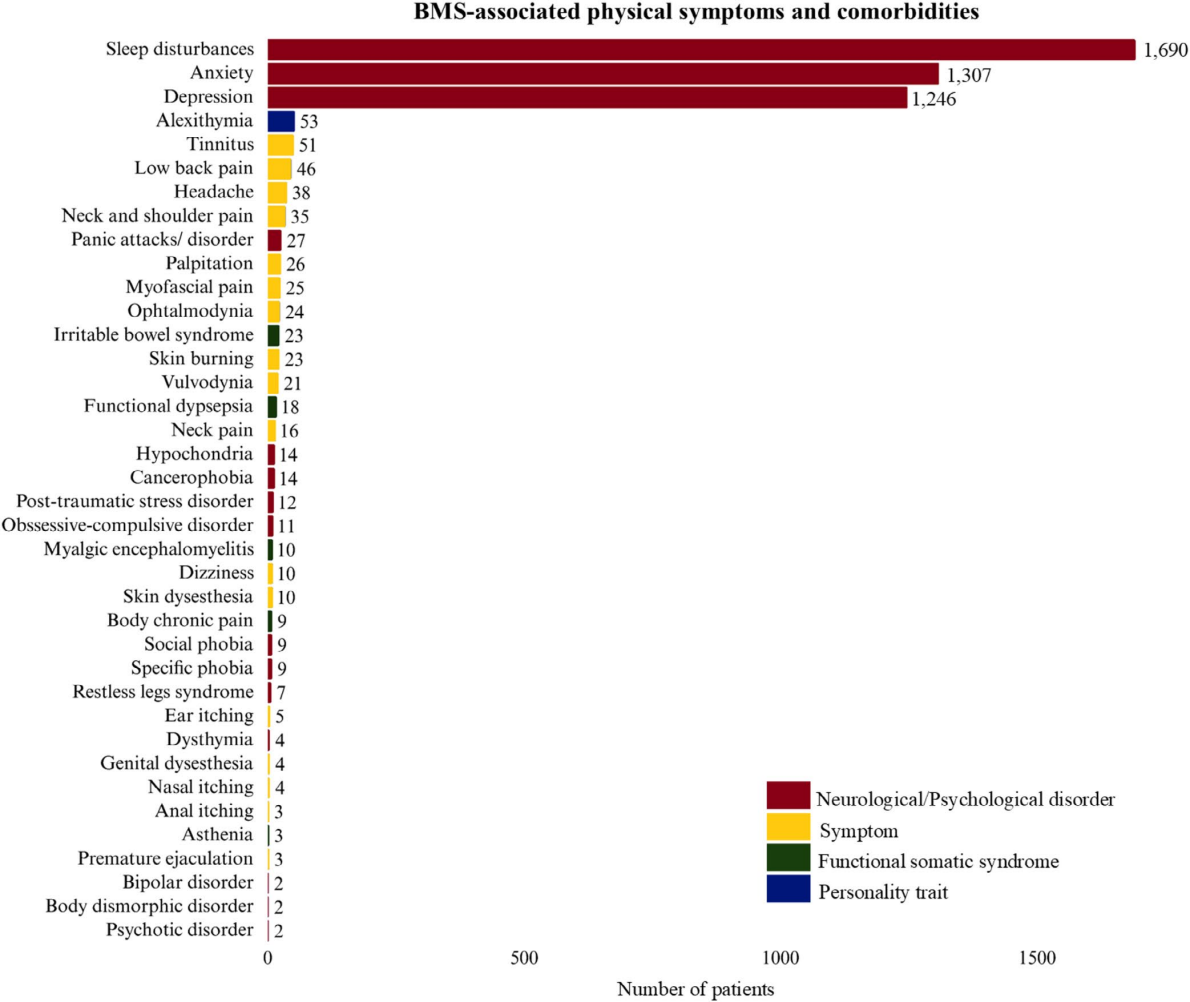
Prevalence of associated extraoral symptoms and comorbid disorders in BMS patients.

Eight hundred sixty‐eight were evaluated for other BMS‐associated extraoral symptoms and comorbidities, encompassing 34 different descriptions. Among these patients, 590 (68%) reported at least one symptom. The most frequent extraoral symptoms were alexithymia (*n* = 53; 6.1%), tinnitus (*n* = 51; 5.9%), low back pain (*n* = 46; 5.3%), headache (*n* = 38; 4.4%), neck and shoulder pain (*n* = 35; 4%), panic attacks/disorder (*n* = 27; 3.1%), palpitations (*n* = 26; 2.9%), myofascial pain (*n* = 25; 2.9%), ophthalmodynia (*n* = 24; 2.7%), irritable bowel disease (*n* = 23; 2.6%), skin burning (*n* = 23; 2.6%), and vulvodynia (*n* = 21; 2.4%). The complete list of reported BMS‐associated extraoral symptoms and comorbid disorders is provided in Figure 2.

## 4 Discussion

This study represented the first systematic review assessing associated extraoral symptoms and comorbid disorders in patients with BMS, providing a broader understanding of the disease's clinical spectrum and highlighting potential areas requiring attention in BMS patient management. Our results reveal that a significant proportion of individuals with BMS experience associated extraoral symptoms and comorbid disorders.

We identified anxiety, sleep disturbances, and depression as the most prevalent co‐occurring psychosocial disorders affecting BMS patients, corroborating previous studies (de Souza et al. 2012; Dibello et al. 2023; Rossella et al. 2022). These associations are supported by both self‐reported screening questionnaires and diagnostic instruments (Calabria et al. 2024; Dibello et al. 2023). Despite the established link between BMS and psychological factors, these elements are frequently overlooked in clinical practice. A history of psychiatric disorders contributes directly to delays in diagnosis, illustrating the condition's complex bidirectional nature. This occurs because clinicians may dismiss symptoms as purely psychological, while patients often misinterpret them as stemming from a physical oral or gastrointestinal problem instead of a psychosomatic origin. The result is a cycle of time‐consuming, ineffective treatments and prolonged diagnostic journeys (Adamo et al. 2024). This mutual misunderstanding highlights a systemic gap in the holistic management of BMS.

The temporal relationship between anxiety, depression, and BMS remains controversial. Given that most studies investigating these factors employ cross‐sectional designs, it is unclear whether these comorbidities contribute to disease development or result from persistent symptoms in BMS patients (Galli et al. 2017; Schiavone et al. 2012). Evidence suggests depression and anxiety may precede a BMS diagnosis. However, diagnostic delays, the often‐invisible nature of patients' complaints, and ineffective treatment strategies further exacerbate the fragile psychological state of BMS patients (Galli et al. 2017). High anxiety and depression levels were also observed in patients with other kinds of chronic conditions, such as atypical facial pain, atypical odontalgia, irritable bowel disease, fibromyalgia, chronic back pain, and chronic fatigue syndrome (Galli et al. 2017). Regardless of causality, their presence alters patients' pain perception, indicates a worse prognosis for BMS, and reduces pharmacological therapy efficacy (Shin et al. 2023). In the absence of a clear diagnosis and a framework to comprehend their condition, patients with a delayed BMS diagnosis are at risk of developing resentment toward the medical system as a whole, creating a significant barrier to establishing an effective patient‐provider relationship. For this reason, psychological assessment must therefore become a mandatory and crucial step in BMS patient management (Adamo et al. 2024).

Sleep disturbances were frequently observed in BMS patients, as shown in our results (Adamo et al. 2018; Galli et al. 2017; Mignogna et al. 2011; Argiuolo et al. 2025). Alterations in sleep quality and duration exhibit a bidirectional relationship with psychological disorders, chronic somatic pain, and mood disorders. Sleep disturbances aggravate symptoms of these conditions, while patients with catastrophic pain experience more severe sleep disruption (Adamo et al. 2018). Since sleep disorders are recognized predictors of depression, their assessment is crucial in managing BMS, particularly in those with no prior history of psychological disorders (Adamo et al. 2018; Johnson et al. 2006). Early identification of sleep disturbances in BMS can prevent severe depression and anxiety onset. Even in patients with BMS experiencing all three conditions, treating residual insomnia is essential to treat depression and prevent relapses (Adamo et al. 2018; Alhendi et al. 2023). Given the high prevalence of sleep disorders in patients with BMS and their significant impact on the condition's severity, alongside the relationship among sleep disturbances, psychological disorders, and chronic pain, patients with BMS must receive proper treatment. This may include referral to sleep medicine specialists, implementation of sleep hygiene practices, or cognitive behavioral therapy for insomnia (Nijs et al. 2018).

Alexithymia emerged as one of the most prevalent BMS‐associated extraoral symptoms and comorbid disorders in this study. Alexithymic individuals struggle to identify feelings and differentiate them from physical sensations of emotional arousal, a characteristic that may partially drive the development and persistence of associated extraoral symptoms and comorbid disorders (Marino et al. 2015). In the general population, the prevalence of alexithymia is approximately 10% (Marino et al. 2015). However, our study observed a lower estimated prevalence of 5.87% among patients with BMS. This discrepancy may indicate underreporting or insufficient investigation of alexithymia in the BMS population, as a previous study reported a 70% prevalence of alexithymia in BMS patients (Jerlang 1997). Furthermore, even a prevalence similar to that of the general population may hold greater clinical significance for BMS patients. The emotional burden associated with the condition's numerous physical and psychological comorbidities can amplify its impact on quality of life. Therefore, future studies are needed to assess the compounded impact of alexithymia and BMS on patient well‐being.

Holistic individualized care and management of patients presenting with BMS necessitates a multidisciplinary approach. The frequent co‐occurrence of these diverse symptoms supports the concept of BMS as a complex disorder that often extends beyond the oral cavity, necessitating a multidisciplinary approach for comprehensive diagnosis and care (Mignogna et al. 2011). Some of these symptoms, including headache, chronic back pain, myalgic encephalomyelitis, and irritable bowel disease, may share underlying central nervous system mechanisms with BMS, as seen in other chronic overlapping pain conditions (Aaron et al. 2000; Aggarwal et al. 2006). Other symptoms may be influenced by psychological comorbidities, such as somatization, which are established features in BMS patients (Mancuso and Berdondini 2005; Mignogna et al. 2011). Moreover, it remains unclear whether these symptoms fall within the spectrum of BMS or represent co‐occurring manifestations. Nevertheless, they should be fully incorporated into the therapeutic management plan (Mignogna et al. 2011).

Since extraoral symptoms and comorbid disorders affect a substantial proportion of BMS patients, their systematic investigation should form an integral part of the clinical assessment. A proactive, multidisciplinary approach that includes referrals to relevant specialists is essential to address the various symptoms that burden BMS patients. Ultimately, recognizing and exploring these co‐occurring symptoms will lead to a more comprehensive understanding of BMS and significantly improve both patient care and quality of life (Santos‐Silva and Villa 2024).

This systematic review identified some limitations. First, numerous studies were excluded for nonadherence to BMS diagnostic criteria established by IHS or ICOP 2020 classifications. Second, many studies did not report data on associated extraoral symptoms and comorbid disorders in absolute or relative frequencies. The absence of this data precluded its inclusion in our analysis and may have led to an underestimation of the true prevalence of these symptoms in the BMS population. Finally, some of the included studies originated from the same research group (Adamo et al. 2018, 2022, 2023, 2024; Canfora et al. 2021 2023; Calabria et al. 2024), suggesting a potential overlap in patient cohorts across publications. To address these limitations, we recommend that future large‐scale cohort, cross‐sectional, and case–control studies consistently apply validated diagnostic criteria and document the presence of these and other extraoral and comorbid conditions on a per‐patient basis. Furthermore, the full spectrum of extraoral symptoms may not be fully captured in the present review, given the variation in assessment methods across the primary studies. To enable standardized documentation and facilitate future comparisons, we recommend adopting the validated instruments outlined in the BMS Research Diagnostic Criteria (RDC/BMS) (Currie et al. 2021). This framework calls for the systematic assessment of comorbid pain conditions such as vulvodynia and somatic symptoms using tools like the PHQ‐15, which measures complaints including headache, back pain, and stomach pain. The incorporation of the PROMIS (Patient‐Reported Outcomes Measurement Information System) Short Form v1.0 for Sleep Disturbance is also advised to ensure a comprehensive evaluation of sleep disorders, a critical aspect for a holistic understanding of BMS. This approach will enable more robust analyses to clarify their associations with BMS.

## 5 Conclusion

A significant proportion of BMS patients experience associated extraoral symptoms and comorbid disorders, including depression, anxiety, sleep disturbances, and other somatic conditions. The high prevalence of these comorbid conditions underscores their clinical significance within the BMS spectrum. However, determining whether they represent true manifestations of the disease itself, rather than merely coincidental occurrences, remains an area for future investigation. Therefore, a comprehensive diagnostic and treatment approach should proactively assess and incorporate the management of these symptoms. Effective care for BMS patients necessitates a multidimensional strategy involving collaboration between oral medicine specialists and other physicians to mitigate the detrimental effects of the disease on patients’ quality of life.

## 6 Other Considerations

This systematic review adhered to the Preferred Reporting Items for Systematic Reviews and Meta‐analyses (PRISMA) Statement (Page et al. 2021) and was registered on the International Prospective Register of Systematic Reviews under the protocol number CRD42025630384.

## Author Contributions

**João Paulo Gonçalves de Paiva:** investigation, writing – original draft, writing – review and editing, visualization, validation, methodology, data curation. **Caique Mariano Pedroso:** writing – review and editing, visualization, validation, methodology, formal analysis, data curation. **Rosa María López‐Pintor Muñoz:** writing – review and editing, visualization, validation. **Milda Chmieliauskaite:** writing – review and editing, visualization, validation. **Alessandro Villa:** writing – review and editing, visualization, validation. **Jacks Jorge:** writing – review and editing, visualization, validation. **Alan Roger Santos‐Silva:** writing – review and editing, visualization, validation, methodology, conceptualization, supervision, data curation.

## Conflicts of Interest

The authors declare no conflicts of interest.

## Supporting information


**Data S1:** Supporting Information.

**Table S1:** Full search strategies used in each scientific database.

**Table S2:** Full data of BMS patients included in this systematic review.

**Table S3:** Critical appraisal of case–control, cohort, and cross‐sectional studies included in this study.

## Data Availability

The data that support the findings of this study are available from the corresponding author upon reasonable request.
