# Supplementary material for: Prevalence of Associated Extraoral Symptoms and Comorbidities in Burning Mouth Syndrome Patients: A Systematic Review
Source: Oral Dis. 2025 Nov 15;32(3):675–83. doi: 10.1111/odi.70144 (PMC13125735; doi:10.1111/odi.70144)
Supplement: Supplementary file 1 — Data S1: Supporting Information. [file ODI-32-675-s004.docx]

**COMPLETE DATA OF PRIMARY STUDIES INCLUDED IN THIS SYSTEMATIC REVIEW**

1. **Adamo et al. (2023) - Adamo D, Calabria E, Canfora F, Coppola N, Pecoraro G, D'Aniello L, Aria M, Mignogna MD, Leuci S. Burning mouth syndrome: Analysis of diagnostic delay in 500 patients. Oral Dis. 2024 Apr;30(3):1543-1554. doi: 10.1111/odi.14553. Epub 2023 Mar 13. PMID: 36825392.**

Italy

Cross-sectional study

**PATIENT SAMPLE**

BMS: 500

CONTROL: NA

F: 371

M: 129

Mean age: 64.5±12.94

**HABITS**

Smokers: 130 (26%), Alcohol use: 74 (14.8%)

**DEPRESSION**

BMS: Hamilton Rating Scale for Depression (HAM-D, Median [IQR]): 17 [14-20]

**ANXIETY**

BMS: Hamilton Rating Scale for Anxiety (HAM-A, Median [IQR]): 17 [15-20]

**SLEEP DISTURBANCES**

BMS: Pittsburgh Sleep Quality Index (PSQI) and Epworth Sleepiness Scale (ESS, Median [IQR]): 8 [7.75-10]; 7 [5-9]. Insomnia: 451 (90.2%)

**BMS CLASSIFICATION**

ICOP, 2020

**ASSOCIATED EXTRAORAL SYMPTOMS/COMORBIDITIES**

Irritable bowel syndrome: 8 (9.6%), Tinnitus: 32 (6.4%), opthtalmodynia: 24 (4.8%), skin burning: 23 (4.6%), vulvodynia: 21 (4.2%), funtional dypsepia: 18 (3.6%), tension headache: 16 (3.2%), dizziness: 10 (2%), restless legs syndrome: 7 (1.4%), ear itching: 5 (1%), nasal itching: 4 (0.8%), low back pain: 3 (0.6%), myofascial pain: 3 (0.6%), anal itching: 3 (0.6%), asthenia: 3 (0.6%), premature ejaculation: 3 (0.6%)

**STATISTICAL DATA**

No statistical tests were performed to evaluate the association between "invisible symptoms" and BMS patients

**MAIN CONCLUSION**

The diagnosis of BMS is often delayed, averaging 29 months in southern Italy, due to limited awareness among healthcare providers. This delay leads to misdiagnosis, multiple consultations, and increased socioeconomic costs, while also worsening patients' psychological distress and quality of life. Educational interventions across medical specialties are needed to promote early diagnosis, focusing on intraoral, extraoral, and systemic symptoms. Establishing standardized diagnostic pathways and specialized orofacial pain centers is crucial to improving BMS assessment and management.

1. **Adamo et al. (2023) - Adamo D, Ottaviani G, Canfora F, Leuci S, Coppola N, Pecoraro G, Rupel K, Preda MTB, Vello V, Albert U, Gobbo M, Guarda-Nardini L, Giudice A, Calabria E, Aria M, D’Aniello L, Biasotto M, Mignogna MD. Loneliness during the COVID-19 Pandemic in Patients with Burning Mouth Syndrome: A Multicentric Case-Control Italian Study. Depress Anxiety. 2023;2023:6666741. doi: 10.1155/2023/6666741. Epub 2023 Nov 8.**

Italy

Case-control study

**PATIENT SAMPLE**

BMS: 100

CONTROL: 100

F: 78 (BMS), 70 (CONTROL)

M: 22 (BMS), 30 (CONTROL)

Mean age: 60.8±13.3 (BMS), 60.7±6.51 (CONTROL)

**HABITS**

NA

**DEPRESSION**

BMS: Depression Anxiety Stress Scales-21 (DASS-21, Median [IQR]): 16 [10-24.2]. 49 patients

**ANXIETY**

BMS: Depression Anxiety Stress Scales-21 (DASS-21, Median [IQR]): 16 [10-24.2]. 55 patients;

CONTROL: Depression Anxiety Stress Scales-21 (DASS-21, Median [IQR]): 10 [4-17]. 31 patients

**SLEEP DISTURBANCES**

BMS: Insomnia Severity Index (ISI, Median [IQR]): 6 [3-11]. 44 patients;

CONTROL: Insomnia Severity Index (ISI, Median [IQR]): 5 [2.75-10]. 37 patients

**BMS CLASSIFICATION**

ICOP, 2020

**ASSOCIATED EXTRAORAL SYMPTOMS/COMORBIDITIES**

NA

**STATISTICAL DATA**

A higher median score in DASS-21 in BMS patients than in controls. Higher stress score in BMS patients than in controls

**MAIN CONCLUSION**

The COVID-19 pandemic increased loneliness in BMS patients, exacerbating stress, anxiety, and depression, particularly in older individuals with higher education and lower social support. Loneliness correlates with worse disease outcomes, cognitive decline, and higher healthcare utilization. Clinicians should integrate loneliness screening into BMS assessments and adopt telehealth to maintain communication and provide psychological support. Addressing loneliness is essential beyond the pandemic, requiring ongoing research and targeted interventions to improve the well-being of elderly patients with chronic pain conditions.

1. **Adamo et al. (2017) - Adamo D, Sardella A, Varoni E, Lajolo C, Biasotto M, Ottaviani G, Vescovi P, Simonazzi T, Pentenero M, Ardore M, Spadari F, Bombeccari G, Montebugnoli L, Gissi DB, Campisi G, Panzarella V, Carbone M, Valpreda L, Giuliani M, Aria M, Lo Muzio L, Mignogna MD. The association between burning mouth syndrome and sleep disturbance: A case-control multicentre study. Oral Dis. 2018 May;24(4):638-649. doi: 10.1111/odi.12807. Epub 2018 Mar 13. PMID: 29156085.**

Italy

Case-control study

**PATIENT SAMPLE**

BMS: 200

CONTROL: 200

F: 163 (BMS), 136 (CONTROL)

M: 37 (BMS), 64 (CONTROL)

Mean age: 61.9±12.1 (BMS), 53.9±9.8 (CONTROL)

**HABITS**

NA

**DEPRESSION**

BMS: Hamilton Rating Scale for Depression (HAM-D, Median [IQR]): 13 [8-20]. 132 patients

**ANXIETY**

BMS: Hamilton Rating Scale for Anxiety (HAM-A, Median [IQR]): 16 [9-22]. 200 patients

CONTROL: Depression Anxiety Stress Scales-21 (DASS-21, Median [IQR]): 10 [4-17]. 31 patients

**SLEEP DISTURBANCES**

BMS: Pittsburgh Sleep Quality Index (PSQI): 158 patients

**BMS CLASSIFICATION**

IHS

**ASSOCIATED EXTRAORAL SYMPTOMS/COMORBIDITIES**

NA

**STATISTICAL DATA**

Poor sleep quality (PSQI ≥ 5) was present in 79% (158) of BMS patients with BMS. BMS patients had statistically higher scores in all items of the PSQI and ESS than the healthy controls (p < .001). A depressed mood and anxiety correlated positively with sleep disturbance. The Pearson correlations were 0.570 for the PSQI vs HAM-D (p < .001) and 0.549 for the PSQI vs HAM-A (p < .001). Pain intensity (NRS) poorly correlated to sleep quality; the Pearson correlation was 0.162 for the PSQI vs NRS (p = .021)

**MAIN CONCLUSION**

The BMS patients showed poor sleep quality, and a higher anxiety and depression prevalence, compared with control patients.

1. **Adamo et al. (2022) - Adamo D, Canfora F, Calabria E, Coppola N, Leuci S, Pecoraro G, Cuocolo R, Ugga L, D'Aniello L, Aria M, Mignogna MD. White matter hyperintensities in Burning Mouth Syndrome assessed according to the Age-Related White Matter Changes scale. Front Aging Neurosci. 2022 Sep 1;14:923720. doi: 10.3389/fnagi.2022.923720. PMID: 36118686; PMCID: PMC9475000.**

Italy

Case-control study

**PATIENT SAMPLE**

BMS: 100

CONTROL: 102

F: 76 (BMS), 79 (CONTROL)

M: 24 (BMS), 23 (CONTROL)

Mean age: 65.34±8.14 (BMS)

**HABITS**

Alcohol use: 15 (15%), Smokers: 24 (24%)

**DEPRESSION**

BMS: Hamilton Rating Scale for Depression (HAM-D, Median [IQR]): 18 [14–21]. 100 patients

**ANXIETY**

BMS: Hamilton Rating Scale for Anxiety (HAM-A, Median [IQR]): 18 [15–21]. 100 patients

**SLEEP DISTURBANCES**

BMS: Pittsburgh Sleep Quality Index (PSQI): 86 patients

**BMS CLASSIFICATION**

ICOP, 2020

**ASSOCIATED EXTRAORAL SYMPTOMS/COMORBIDITIES**

NA

**STATISTICAL DATA**

The BMS patients presented statistically significant higher scores on the ARWMCs compared to the controls, especially in the right frontal, left frontal, right parietal-occipital, left parietal-occipital, right temporal and left temporal lobes. Age, a lower educational level, unemployment, essential hypertension, and hypercholesterolemia were correlated to a higher total score on the ARWMCs. No correlation was found with the blood biomarkers, NRS, SF-MPQ, HAM-A, HAM-D, PSQI, and ESS.

**MAIN CONCLUSION**

This study suggests that BMS patients have a high prevalence of WMHs, particularly in frontal, parieto-occipital, and temporal brain regions, potentially leading to premature brain aging. This may worsen pain perception, mood disorders, and increase the risk of neurodegenerative diseases like AD and VaD. Clinicians should assess cardiovascular risk factors and use brain MRIs as diagnostic tools. Treatment should not only focus on pain and mood but also on reversing WMHs by managing cardiovascular risks and promoting healthy lifestyle habits.

1. **Adamo et al. (2017) - Adamo D, Sardella A, Varoni E, Lajolo C, Biasotto M, Ottaviani G, Vescovi P, Simonazzi T, Pentenero M, Ardore M, Spadari F, Bombeccari G, Montebugnoli L, Gissi DB, Campisi G, Panzarella V, Carbone M, Valpreda L, Giuliani M, Aria M, Lo Muzio L, Mignogna MD. The association between burning mouth syndrome and sleep disturbance: A case-control multicentre study. Oral Dis. 2018 May;24(4):638-649. doi: 10.1111/odi.12807. Epub 2018 Mar 13. PMID: 29156085.**

Italy

Case-control study

**PATIENT SAMPLE**

BMS: 200

CONTROL: 200

F: 163 (BMS), 136 (CONTROL)

M: 37 (BMS), 64 (CONTROL)

Mean age: 61.9±12.1 (BMS), 53.9±9.8 (CONTROL)

**HABITS**

NA

**DEPRESSION**

BMS: Hamilton Rating Scale for Depression (HAM-D, Median [IQR]): 13 [8-20]. 132 patients

**ANXIETY**

BMS: Hamilton Rating Scale for Anxiety (HAM-A, Median [IQR]): 16 [9-22]. 200 patients

CONTROL: Depression Anxiety Stress Scales-21 (DASS-21, Median [IQR]): 10 [4-17]. 31 patients

**SLEEP DISTURBANCES**

BMS: Pittsburgh Sleep Quality Index (PSQI): 158 patients

**BMS CLASSIFICATION**

IHS

**ASSOCIATED EXTRAORAL SYMPTOMS/COMORBIDITIES**

NA

**STATISTICAL DATA**

Poor sleep quality (PSQI ≥ 5) was present in 79% (158) of BMS patients with BMS. BMS patients had statistically higher scores in all items of the PSQI and ESS than the healthy controls (p < .001). A depressed mood and anxiety correlated positively with sleep disturbance. The Pearson correlations were 0.570 for the PSQI vs HAM-D (p < .001) and 0.549 for the PSQI vs HAM-A (p < .001). Pain intensity (NRS) poorly correlated to sleep quality; the Pearson correlation was 0.162 for the PSQI vs NRS (p = .021)

**MAIN CONCLUSION**

The BMS patients showed poor sleep quality, and a higher anxiety and depression prevalence, compared with control patients.

1. **Argiuolo et al. (2025) - Argiuolo A, Canfora F, Muzii B, D'Antonio C, D'Auria D, Giudice A, Musella G, D'Aniello L, Aria M, Maldonato NM, Mignogna MD, Adamo D. Inside the Fire. Exploring the Impact of Anxiety, Depression, and Sleep Disturbances on Pain Perception in Burning Mouth Syndrome: A Cross-Sectional Study of 200 Patients. J Oral Pathol Med. 2025 Aug 11. doi: 10.1111/jop.70026. Epub ahead of print. PMID: 40790615.**

Italy

Cross-sectional study

**PATIENT SAMPLE**

BMS: 200

F: 159 (BMS)

M: 41 (BMS)

**HABITS**

Alcohol use: 43 (21.5%), Smokers: 64 (32%)

**DEPRESSION**

BMS: Hamilton Rating Scale for Depression (HAM-D, Median [IQR]): 16 [6]. 72 patients

**ANXIETY**

BMS: Hamilton Rating Scale for Anxiety (HAM-D, Median [IQR]): 16.5 [6]. 83 patients

**SLEEP DISTURBANCES**

BMS: Pittsburgh Sleep Quality Index (PSQI): 179

**BMS CLASSIFICATION**

ICOP, 2020

**ASSOCIATED EXTRAORAL SYMPTOMS/COMORBIDITIES**

NA

**STATISTICAL DATA**

High anxiety and depression levels were associated with poor sleep quality. Anxiety and depression were associated with higher pain scores.

**MAIN CONCLUSION**

Psychological factors significantly impact the severity of BMS symptoms, underscoring the holistic approach in diagnosis and treatment.

1. **Calabria et al. (2024) - Calabria E, Canfora F, Leuci S, Coppola N, Pecoraro G, Giudice A, Antonelli A, Aria M, D'Aniello L, Mignogna MD, Adamo D. Gender differences in pain perception among burning mouth syndrome patients: a cross-sectional study of 242 men and 242 women. Sci Rep. 2024 Feb 9;14(1):3340. doi: 10.1038/s41598-024-53074-4. PMID: 38336850; PMCID: PMC10858236.**

Italy

Cross-sectional study

**PATIENT SAMPLE**

BMS: 484

F: 242 (BMS)

M: 242 (BMS)

**HABITS**

Alcohol use: 15 (15%), Smokers: 24 (24%)

**DEPRESSION**

BMS: Hamilton Rating Scale for Depression (HAM-D, Median [IQR]): 17 [14-20]): 315 patients

**ANXIETY**

BMS: Hamilton Rating Scale for Anxiety (HAM-A): 324 patients

**SLEEP DISTURBANCES**

BMS: Pittsburgh Sleep Quality Index (PSQI): 349

**BMS CLASSIFICATION**

ICOP, 2020

**ASSOCIATED EXTRAORAL SYMPTOMS/COMORBIDITIES**

NA

**STATISTICAL DATA**

Male BMS patients had a higher proportion of habitual alcohol consumption compared to females. Positive correlation between NRS and TPR-I scores and HAM-A, HAM-D, PSQI and ESS in both groups

**MAIN CONCLUSION**

This research explores the complex factors influencing BMS symptoms across genders. Clinical manifestations, pain severity, and psychological profiles do not significantly differ between men and women, suggesting shared underlying causes, especially in older patients. Sociodemographic factors like education, employment, BMI, and alcohol use affect pain perception in both genders. Lower education and unmarried status are linked to higher pain severity in women, while alcohol use and depression worsen pain quality in men. These findings suggest tailored lifestyle interventions targeting factors like BMI, alcohol, and smoking could improve BMS management.

1. **Canfora et al. (2021) - Canfora F, Calabria E, Cuocolo R, Ugga L, Buono G, Marenzi G, Gasparro R, Pecoraro G, Aria M, D'Aniello L, Mignogna MD, Adamo D. Burning Fog: Cognitive Impairment in Burning Mouth Syndrome. Front Aging Neurosci. 2021 Aug 12;13:727417. doi: 10.3389/fnagi.2021.727417. PMID: 34475821; PMCID: PMC8406777.**

Italy

Case-control study

**PATIENT SAMPLE**

BMS: 40

CONTROL: 40

F: 30 (BMS), 30 (CONTROL)

M: 10 (BMS), 10 (CONTROL)

Mean age: 65.6±8.6 (BMS), 63.7±9.5 (CONTROL)

**HABITS**

BMS: Smokers: 10 (25%), Alcohol use: 8 (20%), CONTROL: Smokers: 8 (20%), Alcohol use: 1 (2.5%)

**DEPRESSION**

BMS: Hamilton Rating Scale for Depression (HAM-D, Median [IQR]): 18 [13.25–27.75]. 39 patients

CONTROL: Hamilton Rating Scale for Depression (HAM-D): 5 patients

**ANXIETY**

BMS: Hamilton Rating Scale for Anxiety (HAM-A, Median [IQR]): 17 [15–21.5]. 40 patients

CONTROL: Hamilton Rating Scale for Anxiety (HAM-A): 7 patients

**SLEEP DISTURBANCES**

BMS: Pittsburgh Sleep Quality Index (PSQI, Median [IQR]): 8.50 [4.28–11]. 30 patients

**BMS CLASSIFICATION**

ICOP, 2020

**ASSOCIATED EXTRAORAL SYMPTOMS/COMORBIDITIES**

NA

**STATISTICAL DATA**

Patients with BMS had impairments in most cognitive domains compared with controls (p < 0.001**) except in RAVLT and CGD. The HAM-D, HAM-A, PSQI, ESS, SF-36, VAS, T-PRI, BPI and PD-Q scores were statistically different between BMS patients and controls (p < 0.001**) the WMCs frequency and ARWMC scores in the right temporal (RT) and left temporal (LT) lobe were higher in patients with BMS (p = 0.023*).

**MAIN CONCLUSION**

BMS is associated with a higher decline in cognitive functions, particularly attention, working memory, and executive functions, but other functions such as praxis-constructive skills and verbal memory are preserved. The early identification of CI and associated factors may help clinicians to identify patients at risk of developing time-based neurodegenerative disorders, such as Alzheimer's disease (AD) and vascular dementia (VD), for planning the early, comprehensive, and multidisciplinary assessment and treatment.

1. **Canfora et al. (2023) - Canfora F, Calabria E, Pecoraro G, Leuci S, Coppola N, Mazzaccara C, Spirito F, Aria M, D'Aniello L, Mignogna MD, Adamo D. Prevalence of hypertension and correlation with mental health in women with burning mouth syndrome: A case-control study. Front Cardiovasc Med. 2023 Jan 20;9:969148. doi: 10.3389/fcvm.2022.969148. PMID: 36741839; PMCID: PMC9894887.**

Italy

Case-control study

**PATIENT SAMPLE**

BMS: 250

CONTROL: 250

F: 250 (BMS), 250 (CONTROL)

Mean age: 62.3±11.4 (BMS), 60.8±11.7 (CONTROL)

**HABITS**

BMS: Smoker: 64 (25.6%), alcohol use: 27 (10.8%), CONTROL: Smoker: 52 (20.8%), alcohol use: 47 (18.8%)

**DEPRESSION**

BMS: Hamilton Rating Scale for Depression (HAM-D). 247 patients

CONTROL: Hamilton Rating Scale for Depression (HAM-D): 81 patients

**ANXIETY**

BMS: Hamilton Rating Scale for Anxiety (HAM-A). 246 patients

CONTROL: Hamilton Rating Scale for Anxiety (HAM-A): 89 patients

**SLEEP DISTURBANCES**

BMS: Pittsburgh Sleep Quality Index (PSQI). 226 patients

CONTROL: Pittsburgh Sleep Quality Index (PSQI). 133 patients

**BMS CLASSIFICATION**

ICOP, 2020

**ASSOCIATED EXTRAORAL SYMPTOMS/COMORBIDITIES**

NA

**STATISTICAL DATA**

Patients with BMS had impairments in most cognitive domains compared with controls BMS patients had statistically significantly higher rates of heavy smoking and lower alcohol use rates compared to healthy patients. BMS patients had a higher frequency of oral symptoms than healthy patients. Severe pain was more frequent in BMS patients. The score of SF-MPQ was high in BMS patients. BMS patients presented a higher frequency of depression and sleep disturbances.

**MAIN CONCLUSION**

WBMS showed a higher prevalence of hypertension (HTN) than healthy women (HW), especially among those with lower education, unemployment, and systemic comorbidities. Although the mechanisms remain unclear, genetic, biological, and environmental factors may contribute to both BMS and HTN. This association may accelerate brain aging and disrupt pain processing. WBMS also reported more anxiety, depression, and sleep disturbances. Early identification and management of HTN and mood disorders, along with healthy lifestyle habits, are crucial to improving quality of life and preventing neurological decline.

1. **Chana et al. (2021) - Chana P, Smith JG, Karamat A, Simpson A, Renton T. Catastrophising, pain self-efficacy and acceptance in patients with Burning Mouth Syndrome. J Oral Rehabil. 2021 Apr;48(4):458-468. doi: 10.1111/joor.13136. Epub 2021 Jan 5. PMID: 33368621.**

England

Cross-sectional study

**PATIENT SAMPLE**

BMS: 36

F: 31 (BMS), M: 5 (BMS)

Mean age: 55.1±9.3 (BMS)

**HABITS**

NA

**DEPRESSION**

NA: 7 patients

**ANXIETY**

NA: 5 patients

**SLEEP DISTURBANCES**

NA

**BMS CLASSIFICATION**

ICHD-3

**ASSOCIATED EXTRAORAL SYMPTOMS/COMORBIDITIES**

Body chronic pain: 9, headache: 5

**STATISTICAL DATA**

Pain catastrophizing was elevated in 32% of patients, with significant associations to anxiety, depression, and reduced oral and general quality of life. Low pain self-efficacy was seen in 24%, and over half showed poor pain acceptance. Catastrophizing correlated strongly with depression (r = 0.80) and HRQoL (rho = −0.84). Self-efficacy and acceptance were also strongly linked to depression and HRQoL, with stronger associations than pain severity alone, even after adjustment.

**MAIN CONCLUSION**

WBMS also had higher rates of anxiety, depression, and sleep disturbances. Early intervention and healthy lifestyle promotion are essential to improve outcomes and quality of life in this population.

1. **Dalirsani et al. (2024) - Dalirsani Z, Amirchaghmaghi M, Semnani MM, Talebi M, Hashemy SI, Shakeri MT, Taghizadeh A. Comparison of Salivary Opiorphin in Burning Mouth Syndrome and Healthy Subjects and Its Correlation With Psychiatric Disorders. Clin Exp Dent Res. 2024 Dec;10(6):e934. doi: 10.1002/cre2.934. PMID: 39616519; PMCID: PMC11608505.**

Iran

Case-control study

**PATIENT SAMPLE**

BMS: 28

CONTROL: 40

F: 23 (BMS), 31 (CONTROL)

M: 5 (BMS), 9 (CONTROL)

Mean age: 52.57 ± 9.76 (BMS), 51.28 ± 9.85 (CONTROL)

**HABITS**

NA

**DEPRESSION**

BMS: Hamilton Rating Scale for Depression (HAM-D): 25 patients

**ANXIETY**

BMS: Hamilton Rating Scale for Anxiety (HAM-A): 28 patients

**SLEEP DISTURBANCES**

NA

**BMS CLASSIFICATION**

ICHD-3

**ASSOCIATED EXTRAORAL SYMPTOMS/COMORBIDITIES**

NA

**STATISTICAL DATA**

BMS patients presented higher anxiety and depression rates

**MAIN CONCLUSION**

Opiophin concentrations are correlated with burning sensation severity in BMS patients.

1. **Dugan et al. (2023) - Dugan C, Popescu BO, Țovaru S, Părlătescu I, Musat IA, Dobre M, Ribigan AC, Milanesi E. Neuropsychological assessment of Romanian burning mouth syndrome patients: stress, depression, sleep disturbance, and verbal fluency impairments. Front Psychol. 2023 May 15;14:1176147. doi: 10.3389/fpsyg.2023.1176147. PMID: 37255508; PMCID: PMC10225505.**

Romania

Case-control study

**PATIENT SAMPLE**

BMS: 120

CONTROL: 110

F: 93 (BMS), 82 (CONTROL)

M: 27 (BMS), 28 (CONTROL)

Mean age: 59.07 ± 11.43 (BMS), 60.56 ± 11.71 (CONTROL)

**HABITS**

BMS: Smokers: 39 (32.5%), alcohol users: 39 (32.5%), CONTROL: Smokers: 39 (35.5%), alcohol users: 49 (44.54%)

**DEPRESSION**

BMS: Hospital Anxiety and Depression Scale (HADS): 15 patients

CONTROL: Hospital Anxiety and Depression Scale (HADS): 1 patient

**ANXIETY**

BMS: Hospital Anxiety and Depression Scale (HADS): 2 patients

CONTROL: 0

**SLEEP DISTURBANCES**

BMS: Pittsburgh Sleep Quality Index (PSQI, Median [IQR]): 9.62 ± 3.76 (2–18). 5 patients

**BMS CLASSIFICATION**

ICHD-3

**ASSOCIATED EXTRAORAL SYMPTOMS/COMORBIDITIES**

NA

**STATISTICAL DATA**

Higher depression prevalence in BMS patients. Worse sleep quality in BMS patients

**MAIN CONCLUSION**

The study concludes that Burning Mouth Syndrome (BMS) significantly impacts quality of life, with patients experiencing high stress, depression, sleep disturbances, and verbal fluency challenges. These findings underscore the need for a multidisciplinary approach, combining dental, psychological, and psychiatric expertise for effective diagnosis and management. Comprehensive care addressing psychological, neurological, and psychiatric dimensions is critical to improving outcomes for individuals with BMS.

1. **Freilich et al. (2020) - Freilich JE, Kuten-Shorrer M, Treister NS, Woo SB, Villa A. Burning mouth syndrome: a diagnostic challenge. Oral Surg Oral Med Oral Pathol Oral Radiol. 2020 Feb;129(2):120-124. doi: 10.1016/j.oooo.2019.09.015. Epub 2019 Oct 12. PMID: 31678041.**

United States of America

Cross-sectional study

**PATIENT SAMPLE**

BMS: 120

F: 88 (BMS)

M: 14 (BMS)

**HABITS**

NA

**DEPRESSION**

NA: 51 patients

**ANXIETY**

NA: 61 patients

**SLEEP DISTURBANCES**

NA

**BMS CLASSIFICATION**

ICHD-3

**ASSOCIATED EXTRAORAL SYMPTOMS/COMORBIDITIES**

Panic attacks: 24 (23.5%), post-traumatic stress disorder: 12 (11.9%), obsessive-compulsive disorder: 7 (6.9%), low back pain: 43 (42.2%), neck and shoulder pain: 41 (40.2%), myofascial pain: 22 (21.6%), skin dysesthesia: 10 (9.8%), genital dysesthesia: 4 (3.9%), irritable bowel disease: 15 (14.7%), chronic fatigue syndrome: 10 (9.8%), tinnitus: 19 (18.6%), palpitation: 26 (25.5%)

**STATISTICAL DATA**

NA

**MAIN CONCLUSION**

Patients with BMS experience delay in diagnosis and management despite seeking and receiving professional care. Many undergo unnecessary tests and tend to be misdiagnosed or receive no diagnosis at all. Even those correctly diagnosed with BMS often receive inappropriate or ineffective treatment.

1. **Khawaja, Bavia, and Keith (2020) - Khawaja SN, Bavia PF, Keith DA. Clinical Characteristics, Treatment Effectiveness, and Predictors of Response to Pharmacotherapeutic Interventions in Burning Mouth Syndrome: A Retrospective Analysis. J Oral Facial Pain Headache. 2020 Spring;34(2):157-166. doi: 10.11607/ofph.2180. PMID: 32255581.**

United States of America

Cross-sectional study

**PATIENT SAMPLE**

BMS: 77

F: 56 (BMS)

M: 21 (BMS)

Mean age: 59.9±10.9

**HABITS**

NA

**DEPRESSION**

NA: 28 patients

**ANXIETY**

NA: 45 patients

**SLEEP DISTURBANCES**

NA: 29 patients

**BMS CLASSIFICATION**

ICHD-3

**ASSOCIATED EXTRAORAL SYMPTOMS/COMORBIDITIES**

Chronic headache disorder: 17, chronic neck pain disorder: 10

**STATISTICAL DATA**

Stepwise forward conditional logistic regression analysis suggested that nonconcurrent use of neuropathic medications was a predictor for significant relief of symptoms in patients with primary BMS. Likewise, the model suggested that presence of anxiety disorder was a predictor in patients with secondary BMS.

**MAIN CONCLUSION**

The prevalence of an associated sensory discrepancy was higher in primary BMS. Pharmacologic intervention provided significant relief for approximately half of the patients with primary BMS and nearly one-third of the patients with secondary BMS. Concurrent use of neuropathic medications was a negative predictor, and presence of anxiety disorder a positive predictor, of therapeutic response among patients with primary BMS and secondary BMS, respectively.

1. **Lee and Chon (2018) - Lee YH DDS, PhD, Chon S MD, PhD. Burning mouth syndrome in postmenopausal women with self-reported sleep problems. Cranio. 2020 Jul;38(4):221-232. doi: 10.1080/08869634.2018.1512549. Epub 2018 Aug 31. PMID: 30165803.**

South Korea

Case-control study

**PATIENT SAMPLE**

BMS: 25

F: 25 (BMS)

Mean age: 55.16±9.61

**HABITS**

NA

**DEPRESSION**

NA

**ANXIETY**

NA

**SLEEP DISTURBANCES**

Symptom Checklist-90-Revised (SCL-90R): 15 patients

**BMS CLASSIFICATION**

ICHD-3

**ASSOCIATED EXTRAORAL SYMPTOMS/COMORBIDITIES**

NA

**STATISTICAL DATA**

Patients with sleep problems had higher frequency of pain, depression and anxiety.

**MAIN CONCLUSION**

Multiple factors are related to the developmental mechanisms of BMS. BMS is often accompanied by both physical and psychological conditions, such as subjective xerostomia, taste disturbances, and abnormal sensation, as well as hormonal changes after menopause, with depression and anxiety regarded as potential predisposing factors. In the presence of sleep disturbances, the BMS patients suffered dysregulated psychoneuroendocrinologic interactions, which might affect oral BMS symptoms and psychological dimensions and aggravate the severity of the burning sensation in postmenopausal BMS patients.

1. **Leuci (2022) - Leuci S, Coppola N, Adamo D, Crocetto F, Barone B, Baldares S, Canfora F, Mignogna MD. Sexual desire, mood disorders and sleep disturbances in female BMS patients: A controlled study. J Oral Pathol Med. 2022 Sep 24. doi: 10.1111/jop.13362. Epub ahead of print. PMID: 36152003.**

Italy

Case-control study

**PATIENT SAMPLE**

BMS: 50

CONTROL: 50

F: 50 (BMS), 50 (CONTROL)

Mean age: 45.6± 6.7

**HABITS**

BMS: Smokers: 14 (28%), alcohol use: 13 (26%), CONTROL: Smokers: 15 (30%), alcohol use: 6 (12%)

**DEPRESSION**

BMS: Hospital Anxiety and Depression Scale (HADS): 8.04 ± 3.18

**ANXIETY**

BMS: Hospital Anxiety and Depression Scale (HADS): 11.86 ± 2.85

**SLEEP DISTURBANCES**

BMS: Pittsburgh Sleep Quality Index (PSQI, Median [IQR]): 9.04 ± 2.63. 47 patients

**BMS CLASSIFICATION**

ICHD-3

**ASSOCIATED EXTRAORAL SYMPTOMS/COMORBIDITIES**

NA

**STATISTICAL DATA**

A total of 50 BMS women and 50 healthy controls were enrolled. Compared with the controls, the BMS patients showed higher scores in the NRS (7,81 ± 1,71 vs. 0,14 ± 0.40; p < 0.0001), TPR-I (10,50 ± 4,86 vs. 0,36 ± 1,06; p < 0.0001), HADS-A (11,86 ± 2,85 vs. 3,90 ± 2,81; p < 0.0001), HADS-D (8,04 ± 3,18 vs. 1,42 ± 1,86; p < 0.0001) and PSQI (9,04 ± 2,62 vs. 4,64 ± 3,27; p < 0.0001). The mean SDI in the study group was significantly lower compared to healthy controls (32,36 ± 14,45 vs. 69,70 ± 19,94; p < 0.0001). No correlation was found between SDI and others items explored

**MAIN CONCLUSION**

In line with previous studies, anxiety, depression and sleep disturbances are more common in BMS patients than in healthy population. This pilot study demonstrates for the first time an association between BMS and low sexual desire.

1. **Marino (2015) - Leuci S, Coppola N, Adamo D, Crocetto F, Barone B, Baldares S, Canfora F, Mignogna MD. Sexual desire, mood disorders and sleep disturbances in female BMS patients: A controlled study. J Oral Pathol Med. 2022 Sep 24. doi: 10.1111/jop.13362. Epub ahead of print. PMID: 36152003.**

Italy

Case-control study

**PATIENT SAMPLE**

BMS: 58

CONTROL: 58

F: 46 (BMS), NA (CONTROL)

M: 12 (BMS), NA (CONTROL)

Mean age: 65.6±10.5

**HABITS**

NA

**DEPRESSION**

BMS: Montgomery and Asberg Depression Rating Scale (MADRS): 7.8±5.1. 57 patients

**ANXIETY**

BMS: Hospital Anxiety and Depression Scale (HADS): 23.5±8.2. 28 patients

**SLEEP DISTURBANCES**

NA

**BMS CLASSIFICATION**

ICHD-3

**ASSOCIATED EXTRAORAL SYMPTOMS/COMORBIDITIES**

Alexithymia: 53 (BMS), Alexithymia: 19 (CONTROL);

**STATISTICAL DATA**

Fifty-eight BMS subjects (46 females and 12 males) had a mean TAS-20 score significantly higher when compared to controls (p<0.001; r=0.72), corresponding to an occurrence rate of alexithymic traits of 79.3 versus 6.9 %. Alexithymic traits inBMS subjectswere just related to depressive traits (p=0.02; ρ=0.31).

**MAIN CONCLUSION**

The high occurrence of alexithymia in BMS is an adjunctive issue in favor of its multifactorial pathogenesis, with a not negligible role for somatization.

1. **De Pedro (2020) - de Pedro M, López-Pintor RM, Casañas E, Hernández G. General health status of a sample of patients with burning mouth syndrome: A case-control study. Oral Dis. 2020 Jul;26(5):1020-1031. doi: 10.1111/odi.13327. Epub 2020 Apr 17. PMID: 32153093.**

Spain

Case-control study

**PATIENT SAMPLE**

BMS: 20

CONTROL: 40

F: 16 (BMS), 32 (CONTROL)

M: 4 (BMS), 8 (CONTROL)

Mean age: 63.95 ± 13.32

**HABITS**

BMS: Smokers: 2 (10%), alcohol user: 6 (15%), CONTROL: Smokers: 1 (2.5%), alcohol user: 0

**DEPRESSION**

BMS: International Classification of Diseases (ICD-11): 9 patients

CONTROL: International Classification of Diseases (ICD-11): 0 patients

**ANXIETY**

BMS: International Classification of Diseases (ICD-11): 6 patients

CONTROL: International Classification of Diseases (ICD-11): 2 patients

**SLEEP DISTURBANCES**

NA

**BMS CLASSIFICATION**

ICHD-3

**ASSOCIATED EXTRAORAL SYMPTOMS/COMORBIDITIES**

NA

**STATISTICAL DATA**

BMS patients had statistically significant differences in mental, behavioural, and neurodevelopmental disorders compared to controls. Controls had significantly better general health compared to BMS. OHIP-14 score was high in BMS compared to controls. BMS showed worsened quality of sleep than controls. Regarding the patients’ psychological status, BMS patients obtained worse results than controls in 8 of the 9 sections of the SCL-90-R

**MAIN CONCLUSION**

BMS patients of the present study presented a significantly worsened overall health status than controls, suffering more illnesses; consuming more medication; and presenting worse general quality of life, worse oral quality of life, greater xerostomia, worse quality of sleep and greater degree of psychological disorders.

1. **Rossella et al (2022) - Rossella I, Alessandro V, Naman R, Gary K, Hervé SY. Topical clonazepam for burning mouth syndrome: Is it efficacious in patients with anxiety or depression? J Oral Rehabil. 2022 Jan;49(1):54-61. doi: 10.1111/joor.13275. Epub 2021 Nov 12. PMID: 34716948.**

United States of America

Cross-sectional study

**PATIENT SAMPLE**

BMS: 82

F: 65 (BMS)

M: 17 (BMS)

Mean age: 65.1±20.5

**HABITS**

NA

**DEPRESSION**

BMS: NA: 46 patients

**ANXIETY**

BMS: NA: 46 patients

**SLEEP DISTURBANCES**

NA

**BMS CLASSIFICATION**

ICHD-3

**ASSOCIATED EXTRAORAL SYMPTOMS/COMORBIDITIES**

NA

**STATISTICAL DATA**

Among the entire cohort, the median symptoms intensity score at baseline was 4.5 and 3.0 at first follow-up, a statistically significant improvement (p < .001; 95% CI). Among the subjects with anxiety/depression and those who were prescribed systemic psychogenic medications, the median symptoms’ intensity score at baseline was 5.0 and 3.0 at first follow-up, a statistically significant improvement (p < .001; 95% CI). Among those without anxiety/depression, the symptoms’ intensity score at baseline was 4.0 and 2.0 at first follow-up, statistically significant improvement (p < .05; 95% CI). The median symptoms’ intensity score for those who were not on any psychogenic medications at baseline was 4.0 and 2.0 at first follow-up, a statistically significant improvement (p < .001; 95% CI)

**MAIN CONCLUSION**

Clinicians are encouraged to prescribe topical clonazepam for BMS regardless of concomitant use of systemic psychogenic medications or comorbid mood disorders as it is an efficacious management approach in the presence or absence of these potentially complicating factors.

1. **Shin et al (2022) - Shin HI, Bang JI, Kim GJ, Kim MR, Sun DI, Kim SY. Therapeutic effects of clonazepam in patients with burning mouth syndrome and various symptoms or psychological conditions. Sci Rep. 2023 May 4;13(1):7257. doi: 10.1038/s41598-023-33983-6. PMID: 37142613; PMCID: PMC10160112.**

South Korea

Cross-sectional study

**PATIENT SAMPLE**

BMS: 41

F: 37 (BMS)

M: 4 (BMS)

Mean age: 66.5 ± 7.92

**HABITS**

NA

**DEPRESSION**

BMS: Beck Depression Inventory (BDI): 31 patients

**ANXIETY**

BMS: NA: State-Trait Anxiety Inventory (STAI): 41 patients

**SLEEP DISTURBANCES**

NA

**BMS CLASSIFICATION**

ICHD-3

**ASSOCIATED EXTRAORAL SYMPTOMS/COMORBIDITIES**

NA

**STATISTICAL DATA**

Patients with burning and taste disturbances reported significant improvement in pain after treatment.

**MAIN CONCLUSION**

A large proportion of BMS patients also experience xerostomia, psychogenic symptoms, and taste change. Clonazepam significantly reduced burning pain in BMS patients with taste disturbances, but no significant reduction in burning pain was found in BMS patients with xerostomia or psychogenic traits only. Such information can be used in BMS patient counseling and the prediction of therapeutic outcomes with clonazepam treatment.

1. **Souza et al (2012) - de Souza FT, Teixeira AL, Amaral TM, dos Santos TP, Abreu MH, Silva TA, Kummer A. Psychiatric disorders in burning mouth syndrome. J Psychosom Res. 2012 Feb;72(2):142-6. doi: 10.1016/j.jpsychores.2011.11.008. Epub 2011 Dec 6. PMID: 22281456.**

Brazil

Case-control study

**PATIENT SAMPLE**

BMS: 30

CONTROL: 31

F: 29 (BMS), 30 (CONTROL)

M: 1 (BMS), 1 (CONTROL)

Mean age: 63.8 ± 11.8

**HABITS**

BMS: Smokers: 2 (3.27%)

CONTROL: Smokers: 3 (4.91%)

**DEPRESSION**

BMS: MINI-PLUS: 14 patients

CONTROL: MINI-PLUS: 4 patients

**ANXIETY**

BMS: MINI-PLUS: 11 patients

CONTROL: MINI-PLUS: 3 patients

**SLEEP DISTURBANCES**

NA

**BMS CLASSIFICATION**

ICHD-3

**ASSOCIATED EXTRAORAL SYMPTOMS/COMORBIDITIES**

BMS: Dysthymia: 4, bipolar disorder: 1, panic disorder: 2, specific phobia: 9, social phobia: 9, obsessive-compulsive disorder: 4, hypochondria: 14, cancerophobia: 14, body dysmorphic disorder: 2, psychotic disorder: 2

CONTROL: dysthymia: 1, bipolar disorder: 3, agoraphobia: 2, specific phobia: 5, social phobia: 6, obsessive-compulsive disorder: 2, hypochondria: 1, cancerophobia: 2, body dysmorphic disorder: 1, adjustment disorder: 2, tic disorder: 1

**STATISTICAL DATA**

BMS patients showed a higher frequency of depression and anxiety than control

**MAIN CONCLUSION**

Patients with BMS have a particular psychological and psychiatric profile.

1. **Wu et al (2022) - Wu S, Zhang W, Wang X, He C, Yan Z. Challenge in the diagnosis, evaluation, and management of burning mouth sensation: A retrospective cohort study. J Am Dent Assoc. 2022 Sep 24:S0002-8177(22)00461-5. doi: 10.1016/j.adaj.2022.07.014. Epub ahead of print. PMID: 36167586.**

China

Cohort study

**PATIENT SAMPLE**

BMS: 178

CONTROL: 405

F: 155 (BMS), 355 (CONTROL)

M: 23 (BMS), 50 (CONTROL)

Mean age: 53.8±12.5

**HABITS**

BMS: Smokers: 1 (0.56%), alcohol use: 3 (1.69%)

CONTROL: Smokers: 7 (1.73%), alcohol use: 7 (1.73%)

**DEPRESSION**

NA

**ANXIETY**

NA

**SLEEP DISTURBANCES**

NA: 77 patients

**BMS CLASSIFICATION**

ICOP, 2020

**ASSOCIATED EXTRAORAL SYMPTOMS/COMORBIDITIES**

NA

**STATISTICAL DATA**

No significant differences were found for age, sex, clinical characteristics, and 14-item Oral Health Impact Profile Questionnaire scores between BMS and no BMS groups. The χ2 test was used for correlation analysis between patients with BMS and without BMS. There were no significant differences in location of symptoms, altered taste, and xerostomia between the BMS and no BMS group (χ2 = 9.43, P = .49; χ2 = 2.30, P = .13; χ2 = 0.05, P = .82, respectively). Oral candidiasis was the most common oral mucosal lesion in patients without BMS (25.19%), followed by oral lichen planus (12.10%) and fissured tongue (4.94%).

**MAIN CONCLUSION**

The study results implied significance of adopting multidisciplinary management of burning mouth sensation. It is imperative for dentists and physicians to strengthen their collaborative relationships and focus on both systemic and oral conditions in these patients.
