# Supplementary material for: Prevalence of Associated Extraoral Symptoms and Comorbidities in Burning Mouth Syndrome Patients: A Systematic Review
Source: Oral Dis. 2025 Nov 15;32(3):675–83. doi: 10.1111/odi.70144 (PMC13125735; doi:10.1111/odi.70144)
Supplement: Supplementary file 2 — Table S1: Full search strategies used in each scientific database. [file ODI-32-675-s001.docx]

**Table S1.** Full search strategies used in each database.

| **PubMed** | (“Burning mouth syndrome” OR “BMS” OR “Burning mouth syndromes”) AND (“anxiety” OR “depression” OR “dizziness” OR “fatigue” OR “bowel difficulties” OR “sexual dysfunction” OR “pain” OR “cognitive impairments” OR “burning” OR “dysgeusia” OR “itching” OR “foreign body sensation” OR “tingling” OR “dysesthesia” OR “dysosmia” OR “symptoms” OR “invisible symptoms” OR “clinical” OR “clinicopathological” OR “clinicopathologic” OR “clinical symptoms” OR “sleep quality” OR “Dysmorphophobia” OR “tumbness” OR “scalding” OR “odontalgia” OR “globus” OR “intangible”) AND (“Prevalence” "prevalences" OR "frequency" OR "frequencies" OR "epidemiology” OR "epidemiologic" OR "epidemiological" OR "occurrence" OR "occurrences" OR “incidence" OR "incidences”) |
| --- | --- |
| **Scopus** | TITLE-ABS-KEY (“Burning mouth syndrome” OR “BMS” OR “Burning mouth syndromes”) AND (“anxiety” OR “depression” OR “dizziness” OR “fatigue” OR “bowel difficulties” OR “sexual dysfunction” OR “pain” OR “cognitive impairments” OR “burning” OR “dysgeusia” OR “itching” OR “foreign body sensation” OR “tingling” OR “dysesthesia” OR “dysosmia” OR “symptoms” OR “invisible symptoms” OR “clinical” OR “clinicopathological” OR “clinicopathologic” OR “clinical symptoms” OR “sleep quality” OR “Dysmorphophobia” OR “tumbness” OR “scalding” OR “odontalgia” OR “globus” OR “intangible”) AND (“Prevalence” "prevalences" OR "frequency" OR "frequencies" OR "epidemiology” OR "epidemiologic" OR "epidemiological" OR "occurrence" OR "occurrences" OR incidence" OR "incidences”) |
| **Embase** | (‘Burning mouth syndrome’ OR ‘BMS’ OR ‘Burning mouth syndromes’) AND (‘anxiety’ OR ‘depression’ OR ‘dizziness’ OR ‘fatigue’ OR ‘bowel difficulties’ OR ‘sexual dysfunction’ OR ‘pain’ OR ‘cognitive impairments’ OR ‘burning’ OR ‘dysgeusia’ OR ‘itching’ OR ‘foreign body sensation’ OR ‘tingling’ OR ‘dysesthesia’ OR ‘dysosmia’OR ‘symptoms’ OR ‘invisible symptoms’ OR ‘clinical’ OR ‘clinicopathological’ OR ‘clinicopathologic’ OR ‘clinical symptoms’ OR ‘sleep quality’ OR ‘Dysmorphophobia’ OR ‘numbness’ OR ‘scalding’ OR ‘odontalgia’ OR ‘globus” OR ‘intangible’) AND (‘Prevalence’ OR ‘prevalences’ OR ‘frequency’ OR ‘frequencies’ OR ‘epidemiology’ OR ‘epidemiologic’ OR ‘epidemiological’ OR ‘occurrence’ OR ‘occurrences’ OR ‘incidence’ OR ‘incidences’) |
| **Web of Science** | TS=(‘Burning mouth syndrome’ OR ‘BMS’ OR ‘Burning mouth syndromes’) AND (‘anxiety’ OR ‘depression’ OR ‘dizziness’ OR ‘fatigue’ OR ‘bowel difficulties’ OR ‘sexual dysfunction’ OR ‘pain’ OR ‘cognitive impairments’ OR ‘burning’ OR ‘dysgeusia’ OR ‘itching’ OR ‘foreign body sensation’ OR ‘tingling’ OR ‘dysesthesia’ OR ‘dysosmia’OR ‘symptoms’ OR ‘invisible symptoms’ OR ‘clinical’ OR ‘clinicopathological’ OR ‘clinicopathologic’ OR ‘clinical symptoms’ OR ‘sleep quality’ OR ‘Dysmorphophobia’ OR ‘numbness’ OR ‘scalding’ OR ‘odontalgia’ OR ‘globus” OR ‘intangible’) AND (‘Prevalence’ OR ‘prevalences’ OR ‘frequency’ OR ‘frequencies’ OR ‘epidemiology’ OR ‘epidemiologic’ OR ‘epidemiological’ OR ‘occurrence’ OR ‘occurrences’ OR ‘incidence’ OR ‘incidences’) |
| **LILACS** | (“Burning mouth syndrome” OR “BMS” OR “Burning mouth syndromes”) AND (“anxiety” OR “depression” OR “dizziness” OR “fatigue” OR “bowel difficulties” OR “sexual dysfunction” OR “pain” OR “cognitive impairments” OR “burning” OR “dysgeusia” OR “itching” OR “foreign body sensation” OR “tingling” OR “dysesthesia” OR “dysosmia” OR “symptoms” OR “invisible symptoms” OR “clinical” OR “clinicopathological” OR “clinicopathologic” OR “clinical symptoms” OR “sleep quality” OR “Dysmorphophobia” OR “tumbness” OR “scalding” OR “odontalgia” OR “globus” OR “intangible”) AND (“Prevalence” "prevalences" OR "frequency" OR "frequencies" OR "epidemiology” OR "epidemiologic" OR "epidemiological" OR "occurrence" OR "occurrences" OR “incidence" OR "incidences”) |
| **ProQuest** | (“Burning mouth syndrome” OR “BMS” OR “Burning mouth syndromes”) AND (“anxiety” OR “depression” OR “dizziness” OR “fatigue” OR “bowel difficulties” OR “sexual dysfunction” OR “pain” OR “cognitive impairments” OR “burning” OR “dysgeusia” OR “itching” OR “foreign body sensation” OR “tingling” OR “dysesthesia” OR “dysosmia” OR “symptoms” OR “invisible symptoms” OR “clinical” OR “clinicopathological” OR “clinicopathologic” OR “clinical symptoms” OR “sleep quality” OR “Dysmorphophobia” OR “tumbness” OR “scalding” OR “odontalgia” OR “globus” OR “intangible”) AND (“Prevalence” "prevalences" OR "frequency" OR "frequencies" OR "epidemiology” OR "epidemiologic" OR "epidemiological" OR "occurrence" OR "occurrences" OR “incidence" OR "incidences”) |
