# Supplementary material for: Prevalence of Associated Extraoral Symptoms and Comorbidities in Burning Mouth Syndrome Patients: A Systematic Review
Source: Oral Dis. 2025 Nov 15;32(3):675–83. doi: 10.1111/odi.70144 (PMC13125735; doi:10.1111/odi.70144)
Supplement: Supplementary file 4 — Table S3: Critical appraisal of case–control, cohort, and cross‐sectional studies included in this study. [file ODI-32-675-s003.docx]

**Tale S3.** Critical appraisal of case-control, cross-sectional, and cohort studies included in this systematic review.

| **Case-control studies** | | | | | | | | | | | |
| --- | --- | --- | --- | --- | --- | --- | --- | --- | --- | --- | --- |
| **Study** | **Q1** | **Q2** | **Q3** | **Q4** | **Q5** | **Q6** | **Q7** | **Q8** | **Q9** | **Q10** | **Bias risk** |
| Adamo (2023) | N | N | Y | Y | Y | Y | Y | Y | Y | Y | Low |
| Adamo (2022) | Y | Y | Y | Y | Y | Y | Y | Y | Y | Y | Low |
| Adamo (2017) | N | Y | Y | Y | Y | Y | Y | Y | Y | Y | Low |
| Canfora (2021) | Y | Y | Y | Y | Y | Y | Y | Y | Y | Y | Low |
| Canfora (2023) | N | Y | Y | Y | Y | Y | N | Y | Y | N | Moderate |
| Dalirsani (2024) | Y | N | Y | Y | Y | Y | N | N | Y | Y | Moderate |
| Dugan (2023) | Y | Y | N | Y | Y | Y | N | Y | Y | N | Moderate |
| Lee and Chon (2018) | Y | N | N | Y | Y | Y | Y | Y | Y | Y | Low |
| Leuci (2022) | Y | Y | Y | Y | Y | Y | N | Y | Y | Y | Low |
| Marino (2015) | Y | Y | Y | Y | Y | Y | N | Y | Y | Y | Low |
| Pedro (2020) | Y | Y | Y | Y | Y | Y | Y | Y | Y | Y | Low |
| Souza (2012) | Y | Y | N | Y | Y | Y | N | Y | Y | Y | Low |
| Tokura (2015) | Y | Y | Y | Y | Y | Y | Y | Y | Y | Y | Low |
| Q1: 1. Were the groups comparable other than the presence of disease in cases or the absence of disease in controls?; Q2: Were cases and controls matched appropriately?; Q3: Were the same criteria used for identification of cases and controls?; Q4: Was exposure measured in a standard, valid and reliable way?; Q5: Was exposure measured in the same way for cases and controls?; Q6: Were confounding factors identified?; Q7: Were strategies to deal with confounding factors stated?; Q8: Were outcomes assessed in a standard, valid and reliable way for cases and controls?; Q9: Was the exposure period of interest long enough to be meaningful?; Q10: Was appropriate statistical analysis used? | | | | | | | | | | | |

| **Cross-sectional studies** | | | | | | | | | |
| --- | --- | --- | --- | --- | --- | --- | --- | --- | --- |
| **Study** | **Q1** | **Q2** | **Q3** | **Q4** | **Q5** | **Q6** | **Q7** | **Q8** | **Bias risk** |
| Adamo (2023) | Y | Y | Y | Y | Y | N | Y | Y | Low |
| Argiuolo (2025) | Y | Y | Y | Y | Y | Y | Y | Y | Low |
| Calabria (2024) | Y | Y | Y | Y | Y | Y | Y | Y | Low |
| Chana (2020) | Y | Y | Y | Y | N | Y | Y | Y | Low |
| Freilich (2020) | Y | N | Y | Y | N | N | Y | Y | Moderate |
| Khawaja (2020) | Y | Y | Y | Y | Y | N | N | N | Moderate |
| Rossella (2021) | Y | N | Y | Y | Y | N | N | N | High |
| Shin (2023) | Y | Y | Y | Y | Y | N | Y | N | Low |
| Q1: Were the criteria for inclusion in the sample clearly defined?; Q2: Were the study subjects and the setting described in detail?; Q3: Was the exposure measured in a valid and reliable way?; Q4: Were objective, standard criteria used for measurement of the condition?; Q5: Were confounding factors identified?; Q6: Were strategies to deal with confounding factors stated?; Q7: Were the outcomes measured in a valid and reliable way?; Q8: Was appropriate statistical analysis used? | | | | | | | | | |

| **Cohort studies** | | | | | | | | | | | | |
| --- | --- | --- | --- | --- | --- | --- | --- | --- | --- | --- | --- | --- |
| **Study** | **Q1** | **Q2** | **Q3** | **Q4** | **Q5** | **Q6** | **Q7** | **Q8** | **Q9** | **Q10** | **Q11** | **Bias risk** |
| Wu (2023) | N | U | U | Y | N | U | Y | N | N | N | N | High |
| Q1: Were the two groups similar and recruited from the same population?; Q2: Were the exposures measured similarly to assign people to both exposed and unexposed groups?; Q3: Was the exposure measured in a valid and reliable way?; Q4: Were confounding factors identified?; Q5: Were strategies to deal with confounding factors stated?; Q6: Were the groups/participants free of the outcome at the start of the study (or at the moment of exposure)?; Q7: Were the outcomes measured in a valid and reliable way?; Q8: Was the follow up time reported and sufficient to be long enough for outcomes to occur?; Q9: Was follow up complete, and if not, were the reasons to loss to follow up described and explored?; Q10: Were strategies to address incomplete follow up utilized?; Q11: Was appropriate statistical analysis used? | | | | | | | | | | | | |
